# Association between arthropathies and postpartum hemorrhage: a bidirectional Mendelian randomization study

**DOI:** 10.3389/fgene.2024.1448754

**Published:** 2024-12-11

**Authors:** Zhao Wu, Chengyu Yuan, Xue Peng

**Affiliations:** ^1^ Department of Obstetrics and Gynecology, Sichuan Provincial People’s Hospital, University of Electronic Science and Technology of China, Chengdu, China; ^2^ Department of Obstetrics and Gynecology, West China Second University Hospital, Sichuan University, Chengdu, China; ^3^ Key Laboratory of Birth Defects and Related Diseases of Women and Children (Sichuan University), Ministry of Education, Chengdu, China

**Keywords:** Postpartum hemorrhage, arthropathies, Mendelian randomization, causality, enrichment analysis

## Abstract

**Background:**

Research links arthropathies with adverse pregnancy outcomes. This study aims to explore its connection to postpartum hemorrhage (PPH) through Mendelian randomization (MR) analysis.

**Methods:**

The study used GWAS data from the IEU OpenGWAS database for PPH and arthropathies. After selecting instrumental variables, bidirectional MR analysis was conducted using MR-Egger, Weighted median, Simple mode, Weighted mode, and IVW methods. Sensitivity analysis was then performed to assess MR results reliability. Finally, enrichment analysis of genes corresponding to arthropathies SNPs in forward MR was conducted to explore their biological function and signaling pathways.

**Results:**

The forward MR results revealed that arthropathies was causally related to PPH, and arthropathies was a risk factor for PPH. Whereas, there was not a causal relationship between PPH and arthropathies by reverse MR analysis. It illustrated the reliability of the MR analysis results by the sensitivity analysis without heterogeneity, horizontal pleiotropy, and SNPs of severe bias by LOO analysis. Furthermore, a total of 33 genes corresponding to SNPs of arthropathies were obtained, which were mainly enriched in regulation of response to biotic stimulus, spliceosomal snRNP complex and ligase activity in GO terms, and natural killer cell-mediated cytotoxicity in KEGG pathways.

**Conclusion:**

This study supported that arthropathies was a risk factor for PPH, and the pathways involved the genes corresponding to SNPs were analyzed, which could provide important reference and evidence for further exploring the molecular mechanism between arthropathies and PPH.

## 1 Introduction

Postpartum hemorrhage (PPH) remains a significant obstetric complication and a major risk during childbirth. Despite notable improvements in healthcare, which have reduced the maternal mortality rate in China to 3.0 per 1,00,000 live births by 2019 due to obstetric hemorrhage, PPH continues to be a leading cause of maternal death. Globally, it is responsible for about 27.1% of all maternal deaths, underscoring the need for ongoing focus on effective management and preventive strategies (Chen et al., 2021; Say et al., 2014). The challenges in managing PPH stem from various factors, including inadequate preventive strategies, limited early detection capabilities, and delayed intervention, underscoring the need for heightened preparedness against this life-threatening condition (Ende et al., 2021).

Arthropathies refer to a broad category of diseases that affect the joints. This term encompasses a wide range of joint disorders, from mild to severe, and includes conditions with different underlying causes, such as inflammatory, degenerative, infectious, and metabolic (Reinus et al., 2009). The common types of arthropathies include rheumatoid arthritis (RA), ankylosing spondylitis (AS), osteoarthritis (OA), gout, psoriatic arthritis (PsA) and arthritis of connective tissue diseases like systemic lupus erythematosus (SLE), scleroderma, and Sjögren’s syndrome. They have been linked to adverse pregnancy outcomes due to abnormal immune activation and subsequent alterations in platelet or coagulation function during pregnancy (Andreoli et al., 2023). This connection suggests a potential increase in PPH risk, further compounded by the effects of common treatments for joint diseases, such as non-steroidal anti-inflammatory drugs and immune modulators, on coagulation and platelet function (García-Fernández et al., 2021).

While there is a recognized association between joint diseases and adverse pregnancy outcomes, the specific relationship with PPH and the underlying mechanisms warrant further exploration. Traditional approaches to establishing causality, such as randomized controlled trials (RCTs), face limitations related to ethical concerns, methodological constraints, and resource demands (Smith and Ebrahim, 2002). Mendelian randomization (MR), leveraging genetic variations as instrumental variables from large-scale genome-wide association studies (GWAS), emerges as a robust alternative for causal inference, mitigating confounding biases inherent in observational studies (Yang et al., 2023; Zhong et al., 2023). Utilizing GWAS data from the IEU OpenGWAS database, this study employs bidirectional MR analysis to elucidate the genetic underpinnings of the association between arthropathies and PPH, offering novel insights into their interplay and informing future research directions (Ramessur et al., 2024).

## 2 Materials and methods

### 2.1 Data sources and summary

Genome-wide association studies (GWAS) summary data of PPH and arthropathies were downloaded from the Integrative Epidemiology Unit (IEU) OpenGWAS database. The dataset of PPH (finn-b-O15) was comprised of 3,670 cases, 98,626 controls and 16,379,289 single nucleotide polymorphisms (SNPs). The dataset finn-b-M13 for arthropathies contained 71,571 cases, 147,221 controls and 16,380,466 SNPs. Genes associated with arthropathies were selected using broad inclusion criteria to capture SNPs linked with a range of joint disorders, including autoimmune, inflammatory, and degenerative arthropathies.

### 2.2 Data pre-processing

The “extract_instruments” function in R package “TwoSampleMR” (Hemani et al., 2018) was employed to read exposure factors and screen instrumental variables (IVs), which were significantly correlated with exposure factors (Forward MR: *P* < 5 × 10^−8^; Reverse MR: *P* < 5 × 10^−6^). Whereafter, the IVs with linkage disequilibrium were discarded (Forward MR: clump = TRUE, r^2^ = 0.001, kb = 10,000; Reverse MR: clump = TRUE, r^2^ = 0.001, kb = 1,000). The “extract_outcome_data” function in R package “TwoSampleMR” (Hemani et al., 2018) was employed to obtain SNPs associated with exposure factors (proxies = TRUE, rsq = 0.8). Passingly, the exposure factor and outcome were arthropathies and PPH in forward MR analysis, respectively, which were swapped as outcome and exposure in reverse MR analysis.

### 2.3 Bidirectional Mendelian randomization (MR) analysis

The “harmonise_data” function in “TwoSampleMR” (Hemani et al., 2018) was utilized to harmonize the effect equipotential with effect size for follow-up analysis. The “mr” function was combined with five algorithms to execute bidirectional MR analysis, including MR Egger (Bowden et al., 2015), Weighted median (Bowden et al., 2016), Inverse variance weighted (IVW) (Burgess et al., 2015), Simple mode, and Weighted mode (Hartwig et al., 2017), while the result of IVW was decisive. For binary outcomes, the odds ratio (OR) and 95%CI was applied to estimate the degree of causality. In addition, in the MR analysis, we tested the effect of exposures on the outcome. Therefore, we used a *p*-value (*P* < 0.05) as the threshold for statistical significance. The results were exhibited by scatter plot, forest plot and funnel plot. Then we also performed the Heterogeneity, Horizontal pleiotropy and Leave-One-Out (LOO) analysis to determine the reliability of the MR results. Moreover, the “mr_pleiotropy_test” function was used for Horizontal pleiotropy to evaluate the presence of confounding factors in this study. For LOO analysis, the meta effect of the remaining SNPs was calculated through the “mr_leaveoneout” function after stepwise elimination of each SNP.

### 2.4 Functional enrichment of genes corresponding to SNPs

Based on SNPs acquired from forward MR analysis, the eQTLGen database was used to identify genes that influence gene expression via cis-eQTL. In order to explore the biological function and signal pathway of these genes corresponding to SNPs, Gene Ontology (GO) and Kyoto Encyclopedia of Genes and Genomes (KEGG) enrichment analysis was carried out via R package “cluster Profiler” (*P* < 0.05) (Wu et al., 2021). The results of GO and KEGG enrichment were visualized by “ggpubr” and “enrichplot”, respectively.

## 3 Results

### 3.1 Forward MR analysis

#### 3.1.1 Arthropathies was causally associated with an increased risk of PPH

**FIGURE 1:**
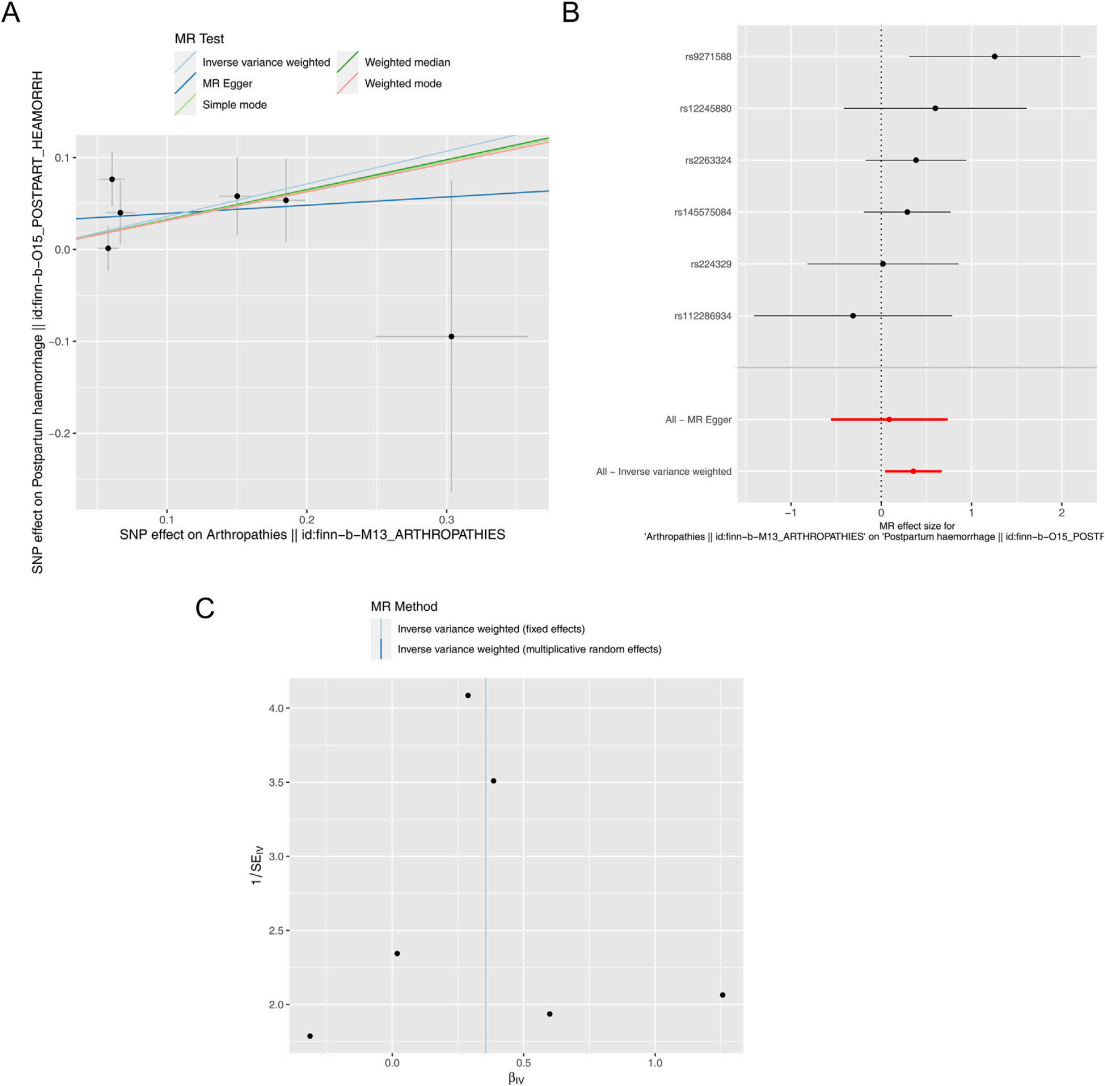
Forward MR analysis. **(A)** Scatter plot of causal relationship between arthropathies and PPH. A regular slope of a line indicates a risk factor, while a negative slope of a line indicates a safety factor. **(B)** Forest plot of causal relationship between arthropathies and PPH. **(C)** Funnel plot of causal relationship between arthropathies and PPH.

After filtrating, a total of six SNPs of arthropathies irrelevant to PPH were acquired as IVs for forward MR analysis (Supplementary Table S1). As presented in Table 1, a causal relationship between arthropathies and PPH was detected by IVW method (*P* = 0.026), and arthropathies was a risk factor for PPH (odds ratio (OR) = 1.428). The scatter plot also revealed that arthropathies was a risk factor for PPH (slope >0) in accordance with the previous MR results (Figure 1A). Obviously, the MR effect size in forest plot exceeded 0, further reflecting that the arthropathies was a risk factor for PPH (Figure 1B). The randomness judgement showed that the forward MR analysis was consistent with Mendel’s second law random grouping (Figure 1C).

**TABLE 1:** Forward MR analysis between arthropathies and PPH.

Outcome	Exposure	Method	nSNP	Pval	Pval
Postpartum haemorrhage id:finn-b-O15_POSTPART_HEAMORRH	Arthropathies id:finn-b-M13_ARTHROPATHIES	MR Egger	6	0.799	1.094 [0.573, 2.086]
Postpartum haemorrhage id:finn-b-O15_POSTPART_HEAMORRH	Arthropathies id:finn-b-M13_ARTHROPATHIES	Weighted median	6	0.065	1.384 [0.980, 1.954]
Postpartum haemorrhage id:finn-b-O15_POSTPART_HEAMORRH	Arthropathies id:finn-b-M13_ARTHROPATHIES	Inverse variance weighted	6	0.026*	1.428 [1.044, 1.952]
Postpartum haemorrhage id:finn-b-O15_POSTPART_HEAMORRH	Arthropathies id:finn-b-M13_ARTHROPATHIES	Simple mode	6	0.248	1.376 [0.853, 2.221]
Postpartum haemorrhage id:finn-b-O15_POSTPART_HEAMORRH	Arthropathies id:finn-b-M13_ARTHROPATHIES	Weighted mode	6	0.182	1.367 [0.920, 2.032]

MR, mendelian randomization; OR, odds ratio; SNP, single-nucleotide polymorphism; Pval, *p* value; * Indicates significant difference (*p* < 0.05).

#### 3.1.2 Reliability of the forward MR results was illustrated by sensitivity analysis

**FIGURE 2:**
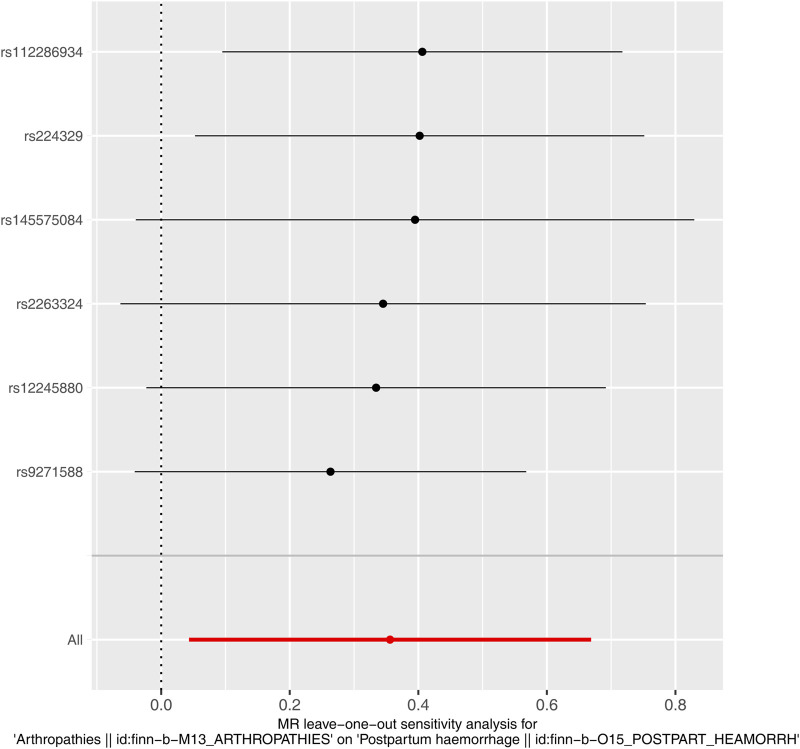
Leave-one-out plot of causal relationship between arthropathies and PPH.

Immediately following the forward MR analysis, the sensitivity analysis was put into effect to evaluate the reliability of forward MR results. The Q_pval values of Heterogeneity test were greater than 0.05 based on MR Egger and IVW methods, suggesting that there was no heterogeneity (Supplementary Table S2). Meanwhile, there was no horizontal pleiotropy (*P* = 0.406), meaning there were no confounding factors (Supplementary Table S3). By eliminating a single SNP, there was no exaggerated influence on the model effect by LOO method (Figure 2). In conclusion, arthropathies was a risk factor for PPH occurrence with the proven reliability.

### 3.2 Reverse MR analysis

#### 3.2.1 PPH was not causally related to arthropathies

**FIGURE 3:**
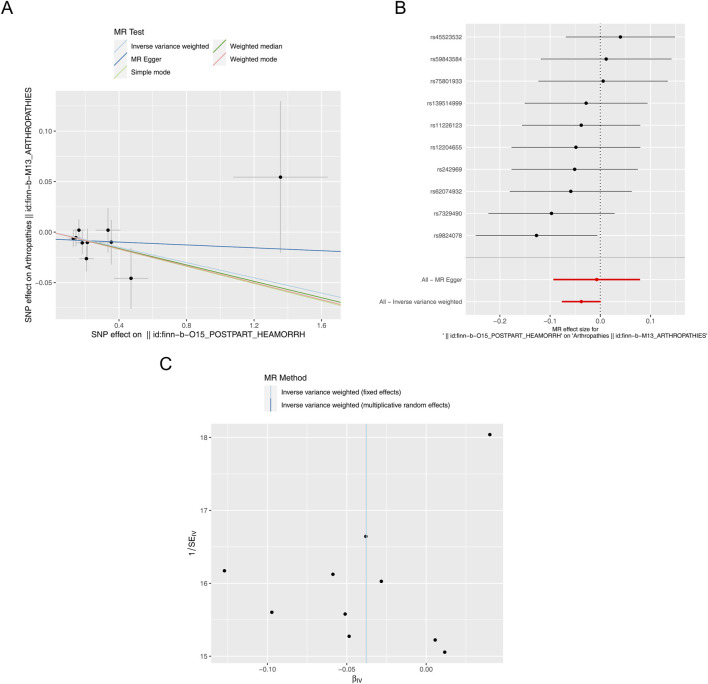
Reverse MR analysis. **(A)** Scatter plot of causal relationship between PPH and arthropathies. A regular slope of a line indicates a risk factor, while a negative slope of a line indicates a safety factor. **(B)** Forest plot of causal relationship between PPH and arthropathies. **(C)** Funnel plot of causal relationship between PPH and arthropathies.

In addition, a total of 10 SNPs of PPH were acquired as IVs after filtrating for reverse MR analysis (Supplementary Table S4). The reverse MR results were presented in Table 2. The *P* values of five methods were greater than 0.05, meaning that PPH was not causally related to arthropathies. The scatter plot also supported this result (Figure 3A). The forest plot was created to evaluate the diagnostic efficiency of each SNP for outcome, suggesting that the overall effect of MR Egger and IVW models was not prominent (Figure 3B). The randomness judgement showed that the reverse MR analysis was consistent with Mendel’s second law random grouping (Figure 3C).

**TABLE 2:** Reverse MR analysis between arthropathies and PPH.

Outcome	Exposure	Method	nSNP	Pval	Pval
Arthropathies id:finn-b-M13_ARTHROPATHIES	Postpartum haemorrhage id:finn-b-O15_POSTPART_HEAMORRH	MR Egger	10	0.875	0.993 [0.911, 1.082]
Arthropathies id:finn-b-M13_ARTHROPATHIES	Postpartum haemorrhage id:finn-b-O15_POSTPART_HEAMORRH	Weighted median	10	0.115	0.960 [0.913, 1.010]
Arthropathies id:finn-b-M13_ARTHROPATHIES	Postpartum haemorrhage id:finn-b-O15_POSTPART_HEAMORRH	Inverse variance weighted	10	0.056	0.963 [0.926, 1.001]
Arthropathies id:finn-b-M13_ARTHROPATHIES	Postpartum haemorrhage id:finn-b-O15_POSTPART_HEAMORRH	Simple mode	10	0.371	0.958 [0.877, 1.047]
Arthropathies id:finn-b-M13_ARTHROPATHIES	Postpartum haemorrhage id:finn-b-O15_POSTPART_HEAMORRH	Weighted mode	10	0.363	0.959 [0.880, 1.045]

MR, mendelian randomization; OR, odds ratio; SNP, single-nucleotide polymorphism; Pval, p value; * Indicates significant difference (*p* < 0.05).

#### 3.2.2 Reliability of the reverse MR results was illustrated by sensitivity analysis

**FIGURE 4:**
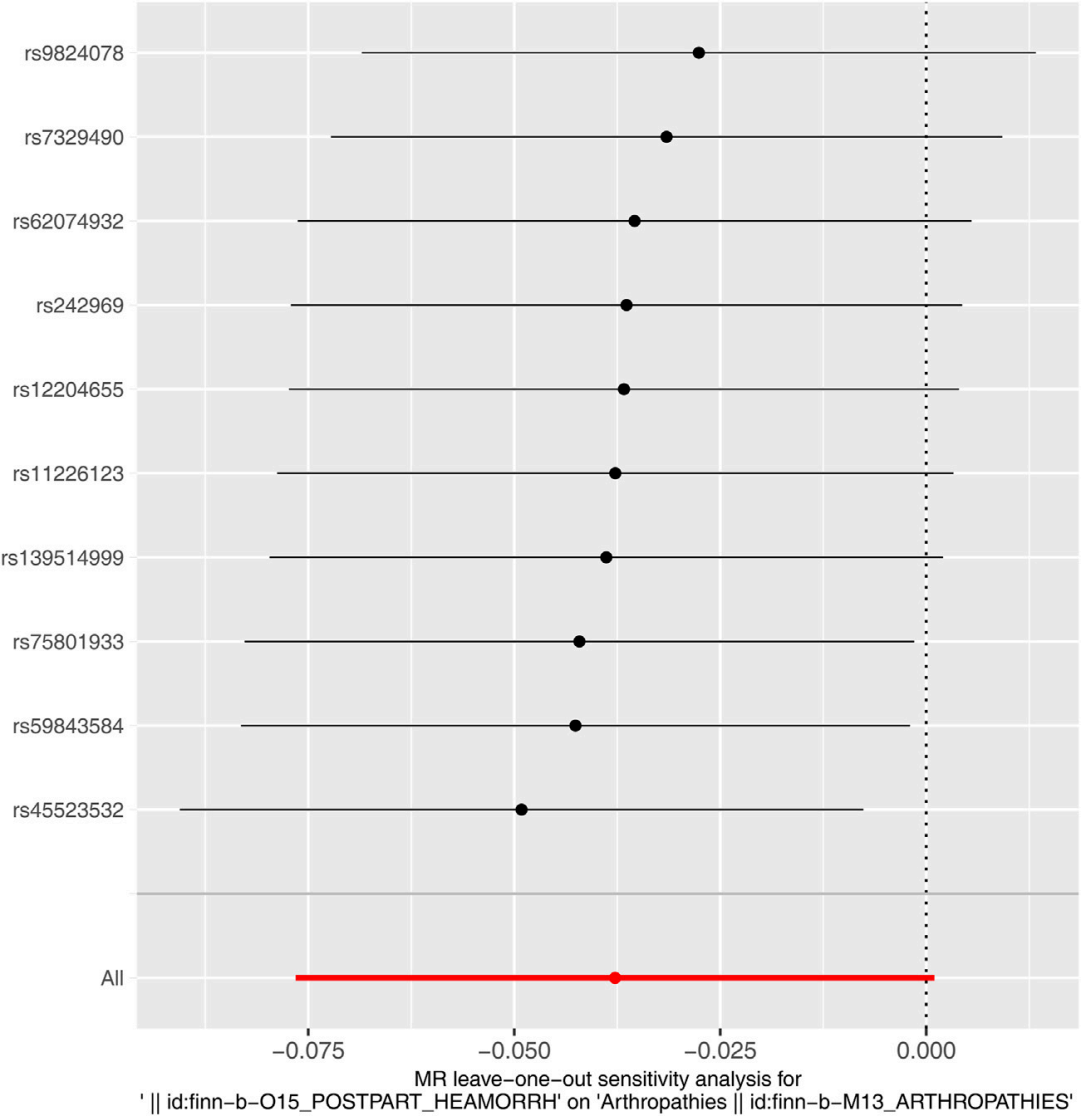
Leave-one-out plot of causal relationship between PPH and arthropathies.

Similarly, immediately following the reverse MR analysis, the sensitivity analysis was put into effect to evaluate the reliability of reverse MR results. The Q_pval values of Heterogeneity test were greater than 0.05 based on MR Egger and IVW methods, suggesting that there was no heterogeneity (Supplementary Table S5). Furthermore, the Pleiotropy test suggested there was no horizontal pleiotropy (*P* = 0.459), meaning there were no confounding factors (Supplementary Table S6), and there were no points of severe bias by LOO method (Figure 4). In conclusion, PPH was not causally influenced on arthropathies.

### 3.3 Enrichment analysis of 33 genes corresponding to SNPs of arthropathies

**FIGURE 5:**
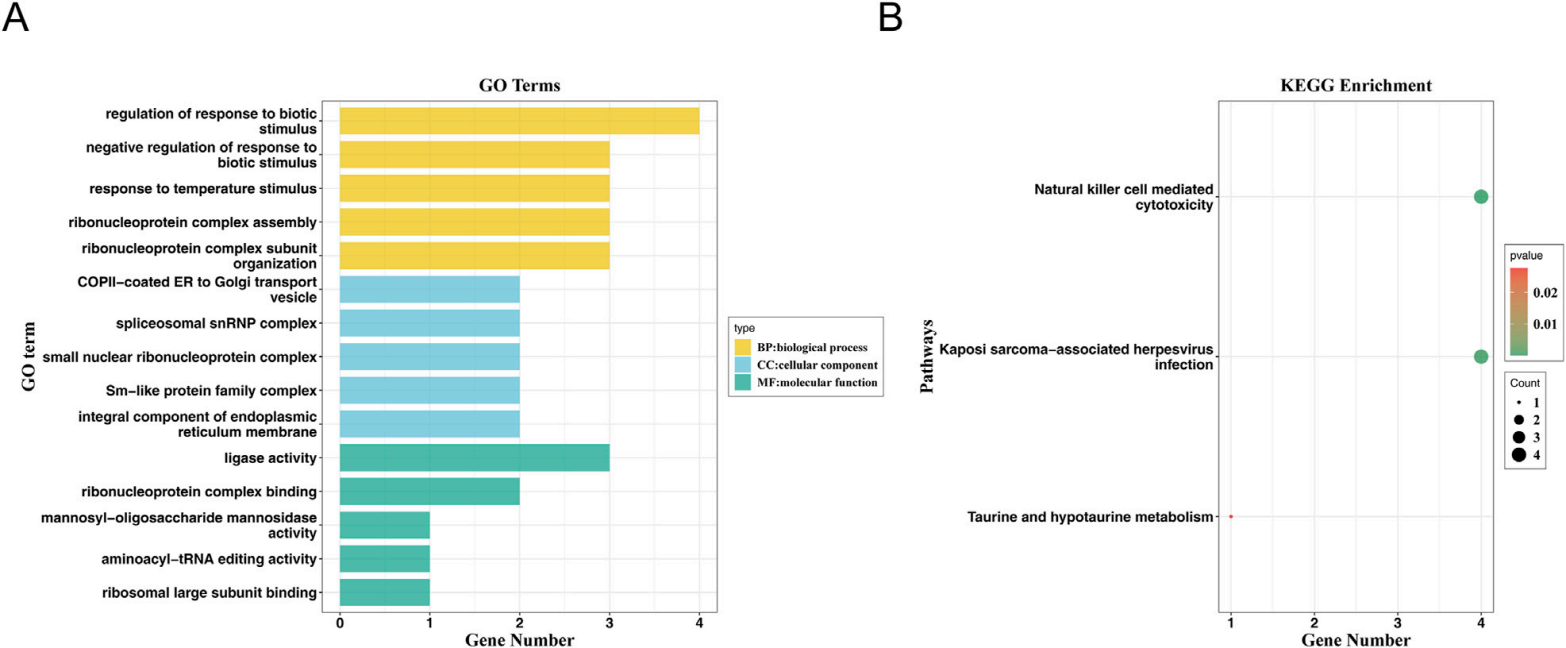
GO and KEGG enrichment analysis. **(A)** GO Function Analysis of Genes. The horizontal axis represents the number of genes enriched in the corresponding pathway, and the vertical axis represents the enriched GO function. **(B)** Bubble diagram of KEGG pathway enrichment of genes.

In total, 33 genes corresponding to six SNPs of arthropathies were obtained for enrichment analysis (Supplementary Table S7). These genes were enriched in 190 GO terms, including 144 in biological process (BP), 25 cellular components (CC) and 21 molecular functions (MF), such as regulation of response to biotic stimulus and ribonucleoprotein (RNP) complex assembly in BP; spliceosomal snRNP complex and small nuclear ribonucleoprotein complex in CC; ligase activity, ribonucleoprotein complex binding, ribosomal large subunit binding and mannosyl-oligosaccharide mannosidase activity in MF (Figure 5A; Supplementary Table S8). Moreover, these 33 genes were markedly enriched in 3 KEGG pathways, including Natural Killer cell mediated cytotoxicity, Taurine and hypotaurine metabolism, and Kaposi sarcoma-associated herpesvirus infection (Figure 5B).

## 4 Discussion

Our investigation into the bidirectional associations between arthropathies and PPH, through a comprehensive Mendelian randomization (MR) analysis utilizing expansive GWAS datasets, predicts a significant genetic predisposition. This predisposition suggests that arthropathies increase the risk of PPH, while no reciprocal genetic evidence was found for PPH influencing the risk of arthropathies. This distinction underlines the critical impact of arthropathies on PPH risk, prompting a closer examination of the underlying biological mechanisms.

Generally, the causes of PPH can be summarized by the four “T’s”: tone (uterine atony), trauma (lacerations, expanding hematomas, or uterine rupture), tissue (retained placental tissue), and thrombin (defects of coagulation) (Bienstock et al., 2021; Committee on Practice Bulletins-Obstetrics, 2017). Abnormal uterine tone is estimated to cause approximately 70% of cases, followed by maternal trauma (approximately 20%), retained placental tissue (approximately 10%), and coagulation deficiencies (<1%) (Bienstock et al., 2021; Committee on Practice Bulletins-Obstetrics, 2017). It is essential to predict the risk and then initiate appropriate interventions (Bienstock et al., 2021; Committee on Practice Bulletins-Obstetrics, 2017).

Arthropathies, such as RA, JIA and SLE, pose unique challenges during pregnancy (Andreoli et al., 2023; Castro-Gutierrez et al., 2022). Women with these conditions are at higher risk for various pregnancy complications, including premature birth, small for gestational age (SGA) infants, disease flares, preeclampsia, miscarriage, and placental abruption et al. (Falcon et al., 2023; Fierro et al., 2023; Ehrmann Feldman et al., 2016; Keum et al., 2024; Zhang et al., 2022). Not all arthropathies behave similarly during pregnancy, disease like SLE, which could be complicated by HELLP syndrome (hemolysis, elevated liver enzymes, and low platelet count), coagulopathies and medications, could lead to PPH (Yan et al., 2024; Sun et al., 2021; Erazo-Martínez et al., 2021; Jiang et al., 2022). The management of JIA during pregnancy is also challenging (Gerosa et al., 2022). A significant concern for women with JIA during the postpartum period is hemorrhage (Chen et al., 2013). Ehrmann Feldman et al. (2017) conducted a population-based cohort study revealing that women with JIA are more likely to experience PPH compared to the controls. These complications highlight the need for effective management of arthropathies during pregnancy involves a multidisciplinary approach, including preconception counseling and close monitoring by rheumatologists and obstetricians (Andreoli et al., 2023). Treatment plans should balance disease control with minimizing fetal risks. Moreover, current evidence recommends the use of prophylactic low-dose aspirin in these patients for the prevention of preeclampsia, and it should be initiated between 12 weeks and 28 weeks of gestation (optimally before 16 weeks) and continued daily until delivery (ACOG Committee Opinion No, 2018).

Limited studies have suggest multiple potential mechanisms through which arthropathies may increase the risk of PPH, including systemic inflammation affecting platelets and vascular health, coagulation disturbance and autoimmune dysregulation, and medication effects. The chronic inflammation characteristic of arthropathies is known to disrupt both the function and number of platelets (Olumuyiwa-Akeredolu et al., 2019). This disruption can lead to a compromised coagulation cascade and hemostatic equilibrium, elevating the risk of bleeding during the postpartum period. In arthropathies, this balance is often skewed towards a pro-hemorrhagic state due to both intrinsic platelet defects and external influences on platelet dynamics (Becker et al., 2018; Tramś et al., 2022).

Beyond the platelet dysfunction, arthropathies are implicated in broader disturbances of the coagulation system, potentially predisposing individuals to either bleeding or thrombotic complications (Wang et al., 2024). The systemic inflammatory milieu can directly impact the endothelium, leading to a reduction in the expression and activity of key anticoagulant pathways and fibrinolytic systems (Vranic et al., 2019). The inflammatory cytokines prevalent in arthropathies, such as tumor necrosis factor-alpha (TNF-α) and interleukin-6 (IL-6), can disrupt the balance between procoagulant and anticoagulant factors (Kang and Kishimoto, 2021; Bouchnita et al., 2017). Commonly, autoimmune conditions can lead to a hypercoagulable state, paradoxically increasing the risk of bleeding by consuming clotting factors and platelets (Borensztajn et al., 2011). As the normal coagulation cascade essential for controlling bleeding during delivery may be impaired, it can significantly delay or prevent the formation of a stable clot, which is a crucial factor in the pathogenesis of PPH (Bank et al., 2023).

The role of inflammation in arthropathies could extend to the vascular endothelium, where chronic inflammatory mediators can degrade endothelial integrity and function (Ho-Tin-Noé et al., 2018). During childbirth, the integrity of the vascular system is paramount in managing the physiological challenges and preventing excessive bleeding. The pre-existing endothelial compromise in individuals with arthropathies may predispose them to hemorrhagic events (Deppermann, 2018). Moreover, autoantibody production and immune complex deposition are hallmark features of arthropathies, where the immune system erroneously targets self-antigens, leading to various clinical manifestations. In the context of pregnancy, such autoimmune activities can significantly impact vascular integrity, coagulation pathways and platelet function, increasing the risk of PPH (Sun et al., 2021).

On the other hand, medications such as low-dose aspirin is commonly used for the prevention of preeclampsia during pregnancy, but it may affect platelet function and increase bleeding risk (ACOG Committee Opinion No, 2018). Similarly, corticosteroids, can influence glucose regulation, wound healing, and blood pressure, which could indirectly increase the risk of PPH (Drechsel et al., 2020). Moreover, biological DMARDs (Disease-Modifying Anti-Rheumatic Drugs) targeting specific inflammatory pathways may also play a role, although their direct effects on pregnancy outcomes and PPH risk are less clear and require further investigation (Drechsel et al., 2020).

In this study, results indicated that arthropathies was genetic-causally related to PPH, and arthropathies was a risk factor for PPH. However, the current information in GWAS databases primarily focuses on the genetic variations related to the overall disease risk, like” arthropathies”, and does not directly provide a clear correspondence between each SNP and specific disease subtypes. To define the specific arthropathies corresponding to the SNPs, we reviewed several relevant studies. rs9271588, this SNP is located in the HLA-DRB1 gene region. Variations in HLA-DRB1 have been associated with several autoimmune conditions, including RA and AS (Tan et al., 2021; Ganjalikhani Hakemi and Nicknam, 2022). rs12245880, a SNP in the IL23R gene, has been linked to AS. Research has demonstrated that variations in the IL23R gene, can influence an individual’s susceptibility to AS (Dong et al., 2013). rs2263324, this SNP is located in the GDF5 gene, which plays a role in bone and cartilage development. Variations in GDF5 have been linked to OA and lumbar disc degeneration (Wang et al., 2023). rs145575084, this SNP has been found possibly related to PsA. It is linked to the IL12B gene, which influences the immune response, contributing to PsA (Filer et al., 2008). rs224329, also situated in the GDF5 gene, this SNP has been associated with chronic low back pain and lumbar disc degeneration (Qi et al., 2023). rs112286934, this SNP is linked to the STAT4 gene. Research indicates that variations in the STAT4 gene are associated with an increased risk of SLE (Liu et al., 2024). However, information on these specific SNPs’ association with arthropathies is limited. Further research is needed to elucidate the potential connections. It is important to note that while certain SNPs are associated with an increased risk of specific arthropathies, they are not definitive predictors. The development of these conditions is influenced by a combination of genetic, environmental, and lifestyle factors. Our study indicates a genetic association between arthropathies and increased PPH risk, seemingly with specific relevance noted for immune-related genes such as HLA and STAT4. However, some identified SNPs, including those associated with degenerative conditions, which seems not logical in any way to induce PPH, require further validation. While this broad selection strategy offered a broad genetic landscape, it also underscores the need for targeted validation studies to confirm the relevance of these findings to PPH risk.

While genetic factors may play a role in individual susceptibility to PPH (Westergaard et al., 2024), the found SNPs of arthropathies have not been identified as significant contributors in current medical literature. To explore the role of genes corresponding to the SNPs, GO functional enrichment and KEGG pathway analyses were performed. In GO analysis, for instance, the biological processes of response to temperature stimulus and RNP complex assembly are fundamental to cellular function. While their direct connections to arthropathies or PPH are not fully elucidated, current research might provide some insights. Temperature sensitivity, mediated by transient receptor potential (TRP) channels, plays a role in pain perception (Ji and Lee, 2021). Dysregulation of these channels may contribute to chronic pain conditions, including arthropathies (Ji and Lee, 2021). Besides, as temperature regulation is vital during labor and delivery, maintaining normothermia is essential for optimal uterine function and reducing PPH risk. Additionally, RNP complex assembly, is crucial for RNA processing and gene regulation (Staley and Woolford, 2009). Given the importance of RNPs in cellular stress responses and inflammation, further investigation into their potential role in arthropathies is warranted, and disruptions in their assembly could potentially impact uterine contractility and hemostasis, indirectly influencing PPH outcomes (McLintock, 2020). This area remains speculative and necessitates further research.

Although none of these genes was directly involved in the coagulation or platelet pathways, the KEGG findings suggest a novel area of exploration in the immunological dysregulation associated with arthropathies and pregnancy, including Natural killer cell mediated cytotoxicity, Taurine and hypotaurine metabolism, and Kaposi sarcoma-associated herpesvirus infection (KSHV). Natural Killer (NK) cells are a type of lymphocyte that plays a vital role in the immune system by targeting and destroying infected or malignant cells. Beyond these roles, NK cells significantly influence inflammatory processes and have been implicated in various autoimmune conditions. In RA, NK cells contribute to joint inflammation and destruction. They can produce pro-inflammatory cytokines like interferon-gamma (IFN-γ) and may exacerbate joint damage (Fathollahi et al., 2021). In systemic JIA, NK cells have been shown to play a role in the disease’s pathogenesis (Put et al., 2017). Recent research has begun to explore their potential involvement in reproductive processes. Studies have shown that uterine NK (uNK) cells can be categorized into different subsets with varying functions during the menstrual cycle and pregnancy (Whettlock et al., 2022). Increased NK cell cytotoxicity has been associated with recurrent spontaneous abortion, suggesting that dysregulation of NK activity can negatively affect placental and vascular integrity. Besides, during pregnancy, a specialized subset of NK cells, known as decidual NK (dNK) cells, accumulates in the uterine lining and is involved in placental development and fetal protection (Xu et al., 2022). While NK cells are primarily recognized for their immune functions, their role in PPH is not well-defined. Some studies suggest that dNK cells contribute to placental vasculature development, and abnormalities in their function could potentially affect uterine contractility and bleeding (Hidaka et al., 1991). However, direct evidence linking NK cell-mediated cytotoxicity to PPH is currently limited.

Disruptions in taurine and hypotaurine metabolism may play a role in the development or progression of arthropathies, possibly through mechanisms involving inflammation and oxidative stress (Yang et al., 2016; He et al., 2023). Moreover, it has been suggested in conditions like intrahepatic cholestasis of pregnancy (ICP), which can have obstetric implications (Tang et al., 2024). However, the direct impact on PPH has not been extensively studied, and current data do not establish a clear connection. Additionally, KSHV infection, while not a common cause of arthropathies, can lead to joint-related symptoms in certain cases, likely through its effects on host cell function and inflammatory pathways (Sychev et al., 2017). While direct evidence of KSHV’s impact on PPH is sparse, the virus’s capacity to induce severe hemorrhagic conditions in other contexts suggests a potential risk factor for similar outcomes in postpartum women, especially those with compromised immune systems (Matsushima et al., 1999; Kulkarni et al., 2023).

This bidirectional MR investigation provides novel genetic insights into the causal link between arthropathies and PPH, revealing potential mechanisms, yet acknowledging inherent limitations such as population homogeneity and the reliance on hypothesis-based instrumental variable validation methods. Although the findings of our study indicate that genetic predictors of arthropathies are notably associated with increased PPH risk, no reciprocal effect was observed, and none of the identified genes directly relate to PPH. This underscores the complexity of immune processes in arthropathies’ pathogenesis and their potential impact on pregnancy outcomes through indirect pathways.

The main concern is that the inclusion of SNPs across all forms of arthropathies presents a limitation. While this approach provided a comprehensive overview, it also encompassed genes related to degenerative conditions (e.g., OA, lumbar disc degeneration) that may lack direct relevance to PPH risk. This selection criterion was an initial exploratory approach, and we acknowledge its limitations in contributing to mechanistic understanding. Future studies should focus on validating genes with clearer immunological relevance, such as HLA and STAT4, and narrowing the inclusion criteria to specific types of arthropathies more plausibly related to PPH risk. This study’s exploratory nature aims to be a preliminary step, aimed at generating causal hypotheses that necessitate experimental follow-up, rather than providing conclusive mechanistic insights.

While experimental validation of this causal relationship is currently unavailable within the scope of this study, our results offer a robust foundation for future targeted *in vitro* and *in vivo* experimental investigations. Expanding the demographic and genetic diversity of study populations will also be crucial in validating and extending these findings to a broader spectrum of individuals affected by arthropathies. In light of these insights, further research could focus on evaluating the biological function of the identified SNPs and genes within relevant immune and coagulation pathways, thus providing more concrete evidence of the mechanisms suggested by our MR analysis. We believe that our reliance on extensive sensitivity analyses and gene expression pathway enrichment sets a strong base for future work, paving the way for experimental immunological research to confirm these associations.

## 5 Conclusion

This study’s Mendelian Randomization analysis elucidates a genetic causal relationship between arthropathies and PPH, with arthropathies identified as a genetic risk factor leading to an increased incidence of PPH. These findings not only contribute to our understanding of the genetic underpinnings linking arthropathies to PPH but also highlight the need for targeted clinical strategies to mitigate PPH risk in patients with arthropathies. Further research, especially in diverse populations and exploring the immunological mechanisms suggested by our findings, is essential for advancing our comprehension of these complex associations.

## Data Availability

The datasets presented in this study can be found in online repositories. The names of the repository/repositories and accession number(s) can be found in the article/Supplementary Material.

## Supplementary material

SUPPLEMENTARY TABLE S1 SNPs information in forward MR analysis.

SUPPLEMENTARY TABLE S2 Heterogeneity test.

SUPPLEMENTARY TABLE S3 Horizontal pleiotropy test.

SUPPLEMENTARY TABLE S4 SNPs information in reverse MR analysis.

SUPPLEMENTARY TABLE S5 Heterogeneity test.

SUPPLEMENTARY TABLE S6 Horizontal pleiotropy test.

SUPPLEMENTARY TABLE S7 Information on 33 genes corresponding to 6 arthropathies of SNP in the enrichment analysis.

SUPPLEMENTARY TABLE S8 GO enrichment information.
